# Nasal septum injury in preterm infants using nasal prongs^1^

**DOI:** 10.1590/0104-1169.3451.2486

**Published:** 2014

**Authors:** Suely de Fátima Santos Freire Bonfim, Maria Gorete Lucena de Vasconcelos, Nayara Francisca Cabral de Sousa, Daiana Vieira Câmara da Silva, Luciana Pedrosa Leal

**Affiliations:** 2 MSc, RN, Unidade Neonatal, Hospital das Clínicas, Universidade Federal de Pernambuco, Recife, PE, Brazil; 3 PhD, Associate Professor, Departamento de Enfermagem, Universidade Federal de Pernambuco, Recife, PE, Brazil; 4 Master's student, Universidade Federal de Pernambuco, Recife, PE, Brazil; 5 RN; 6 PhD, Adjunct Professor, Departamento de Enfermagem, Universidade Federal de Pernambuco, Recife, PE, Brazil

**Keywords:** Continuous Positive Airway Pressure, Premature, Wounds and Injuries, Nursing Care, Nursing

## Abstract

**OBJECTIVE:**

to assess the incidence and risk factors associated with nasal septum injury in premature infants using reused and new nasal prongs.

**METHOD:**

the study was a cohort from an open therapeutic intervention. The sample included 70 infants with a gestational age inferior to 37 weeks, who used nasal prongs and were hospitalized at the neonatal service of a hospital in Recife-PE, in the Northeast of Brazil. The data were collected in patient files through the assessment of the application of the device and of the nasal septum. Multinomial Logistic Regression and Survival analyses were applied.

**RESULTS:**

the incidence of nasal injury corresponded to 62.9%. In the multiple analysis, only the length of the infant's treatment was a determinant factor for the occurrence and severity of the injuries.

**CONCLUSION:**

the type of nasal prong does not serve as a risk factor for the nasal injury. The high incidence of nasal injury indicates the need to adapt the nursing care with emphasis on prevention.

## Introduction

Prematurity stands out as one of the determinant factors of morbidity and mortality in the neonatal period. Appropriate care delivery to infants is one of the risk factors to reduce the child mortality rates^(^ 1 ^)^.

Thanks to the advances in neonatology, an increasing number of premature infants have survived, mainly those of very low weight^(^ 2 ^)^. Non-invasive ventilation throughContinuous Positive Airway Pressure (CPAP) has been used as initial ventilator supportininfants suffering from respiratory discomfort and is part of routine practice at many services^(^ 3 ^)^. Nevertheless, the use of this technology has led to the occurrence of nasal injuries associated with the use of nasal prongs to offer ventilation.

The nasal prong is a device used to offer ventilation. It is adapted to the infant's nostrils and, due to the pressure exerted, can lead to the development of traumas like hyperemia, congestion, lesions, pains, among other complications^(^ 4 ^-^ 6 ^)^. It is the simplest way to offer continuous positive pressure in infants, is less invasive, available in different sizes and made of light and flexible material^(^ 7 ^)^.

The reuse of nasal prongs has been observed in the practice of Brazilian neonatal units, although they are manufactured for single use. This process can lead to color changes and hardening of the device, making it more inflexible and causing nasal injuries in the infants^(^ 8 ^-^ 9 ^)^. An observational study undertaken at a referral unit for high-risk infants showed that 84% of the infants using nasal prongs showed adverse events, 6% of which corresponding to nasal septum injuries^(^ 10 ^)^.

The global prevalence rates of nasal traumas range between 20 and 42.5%^(^ 11 ^)^, reaching 85% to 100% in Brazil^(^ 9 ^,^ 11 ^)^. It is estimated that 88.4% of the neonatal services in the Brazilian Northeast use the non-invasive ventilation method, and that the most used device is the nasal prong^(^ 12 ^)^.

In parallel with this question, another concern is due to the repercussions of these injuries in the family sphere, as the severity of the nasal injury can evolve to the complete loss of the nasal septum, which can disfigure the infant and cause feelings of pain and anguish in the relatives^(^ 13 ^)^.

In that context, in the attempt to improve nursing care for infants, in this study, the following question is raised: what is the incidence of nasal septum injury in preterm infants using new disposable nasal prongs and reused disposable nasal prongs? The obtained results will support the appropriateness of non-invasive ventilation application practices.

Thus, the objective in this study is to assess the incidence and risk factors associated with nasal septum injury in preterm infants using new and reused disposable nasal prongs.

## Method

Analytic and prospective cohort from an open therapeutic intervention, undertaken at the neonatal service of a university hospital in Recife-PE, in the Northeast of Brazil. It is a public institution affiliated with the Ministry of Education, a tertiary referral hospital for care delivery to pregnant women, deliveries and high-risk infants, whose neonatal service offers 13 beds.

The study population consisted of infants with a gestational age inferior to 37 weeks. The sample included all preterm infants admitted between May and November 2012, submitted to non-invasive ventilation (NIV) with nasal prongs, totaling 70 infants.

To randomize the group of infants who received new and reused prongs, routinely adopted at the service, systematic allocation was applied^(^ 14 ^)^, in which the first infant was randomly designated to the device drawn. As from the second, the infants were allocated alternatingly between the new and reused prong groups through their inclusion in the study until the end of the data collection period. This method guarantees that the groups of exposed and non-exposed have the same size^(^ 14 ^)^. In the composition of the groups, eight infants were excluded because they remained in the study for less than 24 hours, resulting in the establishment of two groups: exposed (n=39) using reused prongs and non-exposed (n=31) using new prongs.

The premature infants who received non-invasive ventilation less than 24 hours; suffered from nasal malformations; shock and coagulation disorders and who displayed nasal injury on the occasion of the physical examination upon admission were excluded. The data were collected through the following steps: training of the research team; invitation to the parents for the children's participation, clarifying the different phases of the research; signing of the Free and Informed Consent Term, allocation of the infants to the groups, in which it was evidenced that the reused prongs are used routinely at the service, without additional risks for the infants; consultation of the patient history to collect identification data and sample characteristics; assessment of the nasal area of the infant and monitoring of nursing care delivery.

The research team consisted of eight neonatal nurses, seven of whom were responsible for installing the nasal prong according to the service protocol, and one responsible for the daily assessment of the infant's neonatal area.

To collect the data, three instruments were elaborated: The first addressed the neonatal and treatment-related variables (gestational age, sex, weight at birth, type of prong used and length of NIV). The second refers to the assessment of the infant's nasal septum. The third form was related to the daily verification of the aspects related to the device (identification of standardized brand; prong size appropriate to the infant's weight; care aimed at infants using nasal prongs, such as: use of nasal protection with hydrocolloid; installation of device according to protocol; absence of traction of the prong; fixation of tubes to cap; monitoring of prong position; nasal aspiration with tube caliber appropriate to the infant's weight; nasal humidification; lubrication of nasal prong; routine nasal massage and positioning of the infant using support cushions).

The nurses from the research team performed the daily verification procedures of the assessment instrument and the installation of the device, while the research team and nurses and nursing technicians from the service were responsible for maintaining specific nursing care. Although this treatment is part of the service routine, the entire nursing team was trained before the start of the research. This instrument was submitted to content validation, involving nine expert judges in the theme area of the study, and was considered valid.

To develop the study, the brand of the prongs used, adopted at the place of study, was Hudson-RCI, California, USA. The prong size followed the manufacturer's recommendations, ranging from 0 to 3, according to the infant's weight. Prongs size 00 of the brand Impacto Produtos Médicos e Hospitalares Ltda were used for infants weighing less than 700g at birth, a size the brand Hudson did not attend.

The reused prongs had been subject to plasma peroxide sterilization and maintained their physical integrity and color, a routine procedure at the service. To set a standard, the maximum number of times a device could be reprocessed was set at 20. As this is a single-use device, no specific recommendations were identified in the literature for its reprocessing.

For the daily assessment of the nasal septum integrity, each infant received a form identified with the mother's name and the order number in the study. The two groups received nasal protection with hydrocolloid, which is necessary to prevent nasal injury^(^ 15 ^)^, used routinely at the service. The nursing routines for the application and maintenance of the NIV method were maintained to minimize the interferences of other factors in the dependent variables, as well as any possible harm to the infant.

The nasal septum integrity was assessed in the infants of both groups at two times: upon admission, inspecting the nasal septum before installing the device, guaranteeing the control for the purpose of subsequente comparisons; afterwards, the nasal area was inspected daily. Therefore, the nasal prong had to be removed and reinstalled immediately, after which the appearance of the nasal septum was registered on the form. An observation period of up to 20 days was chosen to monitor the infants, as most cases of nasal trauma due to NIV happen during the first days of treatment, with significant risk after 15 days of use^(^ 11 ^)^.

The nasal injuries were classified in three stages according to the National Pressure Ulcer Advisory Panel (NPUAP) and European Pressure Ulcer Advisory Panel (EPUAP)^(^ 16 ^)^:stage I -intact skin with non-blanchable redness; stage II -presence of ulcer or superficial erosion with partial skin loss andstage III -presence of necrosis and full skin loss.

The infants' characteristics were assessed based on the gestational age, sex and weight at birth. Concerning the treatment, the type of prong and the length of ventilation were assessed.

To analyze the data, the software SPSS (Statistical Package for Social Science), version 17.0 for Windows. Initially, the neonatal variables were subject to descriptive analysis to study the sample characteristics. Sample homogeneity was analyzed by comparing the characteristics between the groups of exposed and non-exposed infants. To verify the normality of the continuous variable, the Kolmogorov-Smirnov Test was applied. For the quantitative research variables, the mean and standard deviations were calculated. To compare the gestational age and weight of the infants in the two groups assessed, Student's t-test was applied.

The categorical variables were analyzed by means of percentage frequencies, and the groups were compared by means of the Chi-Squared and Fisher's Exact Tests. The incidence of nasal septum injury was calculated in the assessed infants and in the groups of exposed and non-exposed infants. In the bivariate analysis, the association among type of prong used, sex, weight at birth, gestational age, length of NIV and the occurrence of the injury was assessed, categorized as: no nasal injury; stage I injury; and stage II or III injury.

For the multivariate analysis, the Multinomial Logistic model was adjusted, considering the following outcome groups: no nasal injury; stage I injury; and stage II or III injury, in order to determine what factors were jointly associated with the nasal septum injury stages. The variables with statistical significance of up to 20% in the bivariate analysis were introduced in the model: gestational age, weight of birth and length of NIV in days. After the initial adjustment, the variable weight at birth showed a lower significance level and was excluded from the model. Next, the model was readjusted and the variable gestational age was removed due to a significance level higher than 5%, leaving only the variable length of treatment.

To verify the level of resistance to the occurrence of nasal septum injury according to the prong type used, sex, gestational age and birth weight related to the length of NIV, survival analysis was applied. This analysis permitted estimated the chances of not developing the nasal septum injury among the study categories. The Log Rank, Breslow and Tarone-Ware Tests were applied to compare the survival according to the factors assessed. All study conclusions were considered, using a significance level of 5%. Approval for the research project was obtained from the Research Ethics Committee at the Health Sciences Center of Universidade Federal de Pernambuco - Brazil.

## Results

The groups of infants according to the prong type used showed no statistically significant differences with regard to sex, weight at birth and gestational age, showing the homogeneity of the groups. In terms of sex, 56.4% of the infants using reused prongs were male and 54.8% of those using new prongs were female.

The mean gestational age of the infants studied was 31.3 weeks (standard deviation, SD = 3.8) for the premature infants using reused prongs and 31.7 weeks (SD = 3.5) for those using new prongs. As regards the weight at birth, the infants showed a mean 1710g (SD = 840g) and 1481g (SD = 573g) in the groups using reused and new prongs, respectively.

The incidence of nasal septum injury corresponded to 62.9%, with 56.4% and 71.8% in the groups using reused and new prongs, respectively. Among the infants with injury, 52.3% showed stage I injuries, 36.4% stage II and 11.3% stage III. In the comparison between the groups, 13.6% of the infants using reused prongs and 9.1% of those using new prongs displayed stage III injuries. No statistically significant differences between the groups were found with regard to the incidence of the injuries (Table 1).

**Table 1:** - Incidenceand stage of nasal septum injury in infants submitted to ventilation using prongs, Recife, PE, Brazil, 2012

Factor assessed		Total n=70			Group under evaluation					p-value
					Reused prong n= 39			New prong n=31		
		n	%		n	%		n	%	
Injury										
	Absent	26	37,1		17	43,6		9	29,0	0,211
	Present	44	62,9		22	56,4		22	71,0	
	Stage EI	23	52,3		12	54,6		11	50,0	*-*
	Stage EII	16	36,4		7	31,8		9	40,9	*-*
	Stage EIII	5	11,3		3	13,6		2	9,1	*-*

In the analysis of the association between the prong type, sex, gestational age, weight at birth and length of NIV and the presence and stages of nasal septum injury, only gestational age and length of NIV showed statistically significant associations. It was verified that, the longer the length of NIV, the higher the incidence of stage II and III injuries, ranging from zero in two days to 66.7% in seven or more days of exposure (Table 2).

**Table 2:** - Association between prong type, sex, gestational age, weight of infant and length of CPAP and the appearance and stage of the injuries. Recife, PE, Brazil, 2012

Factor under evaluation			No injury			EI injury			EII and EIII injury			p-value
			n	%		n	%		n	%		
Prong type												0.436*
	New		9	29.0		11	35.5		11	35.5		
	Reused		17	43.6		12	30.8		10	25.6		
Sex												0.826*
	Female		13	38.2		10	29.4		11	32.4		
	Male		13	36.1		13	36.1		10	27.8		
Gestational age												0.021^†^
	<28 weeks		3	23.1		2	15.4		8	61.5		
	28 to 31 weeks		6	35.3		4	23.5		7	41.2		
	32 weeks or more		17	42.5		17	42.5		6	15.0		
Weight of infant												0.179^†^
		< 1000 g	5	33.3		4	26.7		6	40.0		
		1000 to 1499 g	4	19.0		8	38.1		9	42.9		
		1500 to 2499 g	13	48.1		8	29.6		6	22.2		
		≥ 2500 g	4	57.1		3	42.9		0	0.0		
Length of ventilation												<0.001^†^
	2 days		16	69.6		7	30.4		0	0.0		
	3 to 4 days		6	27.3		11	50.0		5	22.7		
	5 to 6 days		2	15.4		3	23.1		8	61.5		
	7 or more		2	16.7		2	16.7		8	66.7		
												

* Chi-squared test for independence

† Fisher's Exact Test

In the multivariate analysis, only the variable length of NIV showed a statistically significant association with the occurrence of nasal septum injury in the final model. Through this analysis, it was observed that, the longer the length of NIV, the greater the chance of developing stage I or stage II and III injuries when compared to the group of infants without injury (Table 3).

**Table 3:** - Adjusted odds ratio of occurrence of nasal septum injury in infants using prongs. Recife, PE, Brazil, 2012

Factor under Evaluation		Stage I				Stage II and III		
		OR*	CI^†^	p-value		OR*	CI^†^	p-value
Length of NIV								
	2 days	Ref	Ref	Ref		Ref	Ref	Ref
	3 to 4 days	4.19	1.10 – 15.90	0.035		4.77 x 10^8^	‡	§
	5 to 6 days	3.43	0.46 – 25.27	0.227		2.29 x 10^9^	‡	<0.001
	7 or more	2.29	0.27 – 19.66	0.451		2.29 x 10^9^	‡	<0.001

* Odds ratio

† Confidence Interval (95%)

‡ The interval is approximately 108

§ Could not be calculated

The survival analysis of the occurrence of nasal septum injury showed that the group of infants using reused prongs, male, with a gestational age of 32 weeks or more and birth weight of 2,500 g or more showed greater resistance to injuries. Nevertheless, it was verified that, in all factors assessed the survival comparison test was not significant, indicating identical survival rates for nasal septum injuries in these groups.

## Discussion

The study reveals that the prong type used was not a determinant factor for the occurrence and stage of nasal septum injury. The incidence of nasal septum injuries found in the infants corresponded to 62.9%, similar to incidence rates found in the international literature, which have ranged between 40% and 60% in the last three decades^(^ 4 ^)^. In a recent study undertaken in Brazil, the occurrence of nasal septum injury corresponded to 60%, revealing that continuous nasal monitoring care influences the appearance of injuries^(^ 17 ^)^. Statistics can reach up to 100% in Brazil^(^ 9 ^)^.

When considering the severity of the injuries, it is observed that more than 50% of the infants presented stage I injuries. Nevertheless, in the more severe stages, incidence rates of more than 30% are observed in stage II and 10% in stage III. This is a concerning fact in view of the damage observed in these stages, which can range from necrosis to the full loss of the septum. These data differ from results identified in studies that show higher incidences of infants with stage I injuries^(^ 9 ^,^ 11 ^)^.

In the comparison between the groups using reused and new prongs, despite the higher frequency of injuries in infants submitted to new prongs, no statistically significant differences were found. It should be highlighted that, in this study, the number of times the reused prongs were re-sterilized was controlled. Thus, prongs with color changes, hardening of the silicon and cracks were discarded, a procedure that is part of the care taken to prevent nasal septum injuries in premature infants^(^ 8 ^)^, which may have influenced the minimization of the occurrence of injuries in the group of exposed infants.

Similar results were found when assessing the efficacy and problems associated with the application of nasal CPAP, where no statistically significant difference was found in the incidence of redness and nasal bleeding due to the use of new and reused prongs^(^ 8 ^)^.

Another aspect that should be considered was the fact that, in this study, an instrument was used to monitor the nursing care, elaborated based on evidence from the literature about the care recommendations needed to prevent nasal septum injury in infants using prongs. This care applied daily to all infants may have contributed to the lower incidence levels of nasal septum injury.

The factors that were separately associated with the appearance of injuries were the gestational age and the length of NIV using prongs. The increased frequency and severity of the injuries was observed in infants with a gestational age inferior to 28 weeks, reaching 61.5% with stage II and III injuries. These findings are ratified by results found by other authors^(^ 5 ^,^ 18 ^)^, showing that infants of a younger gestational age show a trend towards more severe injuries.

Based on these findings, the immaturity of these premature infants' integumentary system should be noted, with a decreased barrier function of the skin, making it more susceptible to traumas and, consequently, infections^(^ 18 ^)^. Hence, the occurrence of these injuries is directly related to the pressure the device exerts on the septum and nasal columella, due to the reduced local blood irrigation and the consecutive development of trauma^(^ 5 ^,^ 9 ^,^ 11 ^)^. Hence, the non-constant observation of these devices' positioning and stabilization favors the appearance of injuries, demanding immediate intervention from the team for the sake of prevention^(^ 19 ^)^.

As regards the length of ventilation using prongs, it is identified that, the longer the treatment, the more severe the injuries become. Similar results were found in other studies that assessed this association^(^ 11 ^,^ 17 ^-^ 18 ^)^. Hence, in practice and in scientific evidence, the length of treatment is a risk factor for the evolution and severity of the injuries.

In that sense, the longer use of the prong requires effective participation from the nursing team in strictly complying with the care protocol for infants using NIV with prongs. In a recent study, it was observed that most of the injuries due to nasal prongs appear at night, when the surveillance at the neonatal intensive care unit probably decreases. Also, the work overload at some times of the day can reduce the surveillance of the infants using this device^(^ 20 ^)^. This fact entails the need for qualification and appropriate dimensioning of the nursing staff attending to infants.

In this study, although the infants' weight was not associated with the occurrence and severity of the nasal septum injury, a trend towards longer NIV use was observed in lighter infants. None of the premature infants weighing more than 2,500g showed severe injuries. The same result was found in a study that associated the use of masks and nasal prongs with the occurrence of injuries^(^ 5 ^)^. The literature shows that, in infants using short binasal prongs, low birth weight and the length of NIV are directly associated with the development of nasal injuries^(^ 17 ^-^ 18 ^,^ 21 ^)^.

In the multivariate analysis, the gestational age lost its significance, so that only the length of the non-invasive ventilation with prongs remained, independently associated with the occurrence and severity of the injuries. This fact has been confirmed in scientific publications^(^ 11 ^,^ 18 ^)^. It was demonstrated in research to test silicon gel for nasal septum protection that the treatment length represented a risk factor for the development of nasal injuries and that the product tested granted greater protection to the infants studied^(^ 11 ^)^.

In the survival analysis, it was evidenced in this research that premature infants exposed to the use of new prongs present a greater probability of not getting injured during the first days and, as from the third day of treatment, this probability moved to the group using reused prongs, confirming the results found in the multivariate analysis that there is no statistically significant association between the use of new or re-sterilized nasal prongs and the incidence of septum injury, independently of the duration of the NIV.

The fact that male infants with a gestational age of 32 weeks or more and a birth weight of 2,500g or more are more resistant to the occurrence of nasal septum injury did not indicate any difference in survival between the groups. What the gestational age is concerned, however, it should be considered that, besides the infant's cutaneous weakness, the length of the NIV tends to be inversely proportional to the gestational age, so that younger premature infants tend to remain in the treatment longer^(^ 5 ^)^and are exposed to worse injuries, as the injuries appear around the fourth day of treatment.

In fact, perhaps, the control of the number of times the devices are sterilized, due to the need to preserve their consistency, which guarantees their softness^(^ 9 ^)^, in combination with the compliance with the nursing care protocol to prevent nasal septum injuries in premature infants, may be more associated with the drop in the incidence rates of the injuries that the type of prong used in the treatment. This fact entails the need for sensitization and continuous training of the team to monitor the infants, as well as for the early detection of the injuries and measures to prevent their development and worsening^(^ 20 ^)^.

In view of the lack of recent studies on this theme in the literature, the technological evolution at the neonatal services has moved towards the search for devices that minimize the effects on the infant's nostrils. Nevertheless, despite the increasing use of these devices, there are no controlled studies yet that guarantee safety and efficacy in the neonatal patients^(^ 18 ^-^ 19 ^)^.

## Conclusion

This study showed that more than half of the infants using NIV with nasal prongs present nasal septum injuries and that the type of prong used does not represent a determinant factor for the development of these injuries. It was also observed that, the younger the gestational age and the longer the length of treatment, the higher the incidence rate and the more severe the injuries become. In the multivariate analysis, only the length of NIV treatment was a determinant factor for the appearance of the injuries.

The results support attitude changes in the nursing team with a view to compliance and reassessment of routines, based on the evidence found here. Studies are needed to test the use of new devices brought onto the market, in order to guarantee the adoption of increasingly safe practices in neonatal care.
